# Epigenetics in Heart Failure: Role of DNA Methylation in Potential Pathways Leading to Heart Failure with Preserved Ejection Fraction

**DOI:** 10.3390/biomedicines11102815

**Published:** 2023-10-17

**Authors:** Simon W. Rabkin, Chenille N. Wong

**Affiliations:** 1 Department of Medicine, University of British Columbia, Vancouver, BC V6T 1Z4, Canada; 2 Division of Cardiology, University of British Columbia, Vancouver, BC V5Z 1M9, Canada

**Keywords:** DNA methylation, heart failure with preserved ejection fraction, cardiac fibrosis, obesity, cardiac metabolism

## Abstract

This review will focus on epigenetic modifications utilizing the DNA methylation mechanism, which is potentially involved in the pathogenesis of heart failure with preserved ejection fraction (HFpEF). The putative pathways of HFpEF will be discussed, specifically myocardial fibrosis, myocardial inflammation, sarcoplasmic reticulum Ca^2+^-ATPase, oxidative–nitrosative stress, mitochondrial and metabolic defects, as well as obesity. The relationship of HFpEF to aging and atrial fibrillation will be examined from the perspective of DNA methylation.

## 1. Introduction

Despite significant progress in its diagnosis and treatment, heart failure (HF) continues to be associated with a high prevalence and high rates of morbidity and mortality [1]. Heart failure has been categorized into groups based on left ventricular ejection fraction (EF), which includes HF with preserved EF (HFpEF), HF with reduced EF (HFrEF), and HF with mid-range EF (HFmrEF) [2,3]. Heart failure with compromised ejection fraction has usually been attributed to a loss of cardiomyocytes, which occurs from acute processes such as myocardial infarction or myocarditis [2,3]. HFpEF is a complex disease due to its various purported pathophysiologies and subtypes [4,5,6,7] and accounts for approximately half of all HF cases [1]. The search for new avenues to understand and treat heart failure has begun to focus on the role of epigenetic regulation of gene expression and activity in the pathogenesis of heart failure. Epigenetic modifications present clues to the pathogenesis of heart failure, and multiple epigenetic pathways have been proposed that might lead to the development of heart failure (HF) [8,9,10,11,12]. Epigenetic modifications refer to changes in gene regulation that are independent of alterations in DNA sequence, including DNA methylation, ATP-dependent chromatin remodeling, histone modifications, and microRNA mechanisms [13]. A risk model based on both clinical and DNA methylation data outperformed a model based on just clinical data from the electronic health care record for the prediction of the development of HFpEF [14]. This review will focus on epigenetic modifications utilizing the DNA methylation mechanism, which is potentially involved in the pathogenesis of HFpEF. These putative pathways of HFpEF will be discussed: myocardial fibrosis, myocardial inflammation, sarcoplasmic reticulum Ca^2+^-ATPase, oxidative–nitrosative stress, mitochondrial and metabolic defects, as well as obesity. The relationship of HFpEF to aging and atrial fibrillation will be examined from the perspective of DNA methylation.

## 2. DNA Methylation

DNA methylation is catalyzed by a family of DNA methyltransferases that transfer a methyl group to cytosine residues that are followed by guanine (CpG) [15,16]. In the majority of cases, DNA is methylated at the C5 position of cytosine in CpG dinucleotides or CpG sites that tend to be in clusters in the genome called CpG islands [17]. DNA methyltransferase enzymes (DNMTs) include DNMT1, DNMT2, DNMT3A, and DNMT3b [15,16,18]. DNMT3a and DNMT3b are sometimes referred to as *de novo* DNMTs because they can establish a new methylation pattern in unmodified DNA [16]. Mammalian DNA methyltransferases are encoded by their own single gene and consist of catalytic and regulatory regions (except DNMT2) [15]. Non-CpG methylation can occur, but its role is not completely defined [16]. The hypermethylation of a promoter prevents transcription factor binding or recruits repressor complexes, resulting in gene repression, whereas hypomethylation results in gene expression [19].

DNMT3A/3B enzymes are most relevant in the heart because of the low rates of DNA synthesis in adult cardiomyocytes [20]. DNMT3A has been identified as the main cardiac DNMT because of its high transcript abundance in cardiomyocytes relative to the other isoforms [21]. The consequences of DNA methylation include gene silencing or activation, depending on the methylated regions [22].

## 3. DNA Methylation in Cardiomyocytes and the Heart

Genomic regions are differentially methylated during cardiomyocyte development and maturation [23]. *De novo* methylation by DNA methyltransferases 3A/B induces a repression of fetal cardiac genes, including essential components of the cardiac sarcomere [23]. The similarity of the DNA methylation patterns of cardiomyocytes in HF to neonatal methylation patterns suggests that heart failure is associated with a ‘reversion’ to neonatal gene expression patterns in heart failure [23].

In human-induced pluripotent stem cell-derived cardiomyocytes, the knockout of DNMT3A was found to have three main consequences: (i) gene expression changes in contractile proteins; (ii) aberrant activation of the glucose/lipid metabolism regulator peroxisome proliferator-activated receptor gamma; and (iii) hypoxia-inducible factor 1α protein instability that was associated with impaired glucose metabolism and lower glycolytic enzyme expression [21].

Pepin et al. examined DNA methylation in left ventricle tissue obtained from seven patients with end-stage HF (HFrEF) and three donor hearts without heart failure [24]. Hypermethylated promoters were associated with genes involved in oxidative metabolism, specifically acetyl-CoA acetyltransferase 1, isopentenyl-diphosphate Δ-isomerase, farnesyl diphosphate synthase, and 3-hydroxy-3-methylglutaryl-CoA synthase 1. Heart failure was also associated with promoter hypomethylation of enriched glycolytic pathways and anaerobic metabolic processes, including phosphofructokinase (PFKL and PFKP), enolase (ENO1, ENO2, and ENO3), and glyceraldehyde-3-phosphate dehydrogenase (GAPDH) [24]. Supporting the role of DNA methylation in heart failure, there are data that demonstrate that overexpression of DNMT3A reduces the expression of oxidative metabolic genes in H9c2 rat cardiomyoblasts [24]. There is also binding-site competition via hypermethylation of the nuclear respiratory factor 1 (NRF1) motif, an upstream regulator of mitochondrial biogenesis [24].

Liao et al. studied 36 patients with end-stage HF, 12 with ischemic cardiomyopathy (ICM), 24 with non-ischemic dilated cardiomyopathy (NICM), and 7 controls without heart failure [25]. DNA methylation profiling identified 2079 differentially methylated gene (DMP) positions in the myocardium of patients with ICM, of which 625 were hypermethylated and 1454 DMPs were hypomethylated [25]. A total of 261 DMPs were differentially methylated in the myocardium of patients with NICM compared to the controls, of which 117 DMPs were hypermethylated and 144 were hypomethylated [25]. There were common hypermethylated (*n* = 67) and hypomethylated (*n* = 125) sites in ICM and NICM [23]. The T-box transcription factor TBX3 was the only protein-coding gene common to ICM and NICM with DNA hypermethylation and transcriptional downregulation [25]. Interestingly, DNA methylation of transcription factor binding sites is involved in ventricular development [26]. LINC0088 (long intergenic non-protein coding RNA 881), which is abundantly expressed in the adult human heart, is hypermethylated in heart failure, including both ICM and NICM, and is associated with the downregulation of LINC00881 gene expression [23]. Data suggest that it is a regulator of cardiomyocyte calcium cycling and an upstream transcriptional regulator of several key calcium channel and sarcomere organization genes [25].

There are data suggesting that DNMT3a and DNMT3b are not operative in heart failure, as DNMT3a/3b ablation in mice did not increase in heart failure after increased cardiac loading from aortic constriction [20]. Promoters of upregulated genes were largely unmethylated in DNMT3a/3b knockouts compared to control mice [20]. The abundance of human data [24,25], would outweigh the data from that animal model.

While studies have investigated the impact of DNA methylation on cardiac function and heart failure with reduced ejection fraction [20,21,24,25,26,27], the epigenetic role of DNA methylation in the pathogenesis of HFpEF has not been well characterized. An analysis of this subject will address the different putative pathways leading to HFpEF. The objectives of this study were to explore how epigenetic changes related to DNMT3A/B can modulate or induce HFpEF. By doing so, we hoped to clarify how epigenetic mechanisms may be considered in the pathophysiology and future treatment of HFpEF.

## 4. Myocardial Fibrosis

HFpEF is linked to increased myocardial collagen as well as collagen-dependent and titin-dependent stiffness [28,29]. Excess tissue fibrosis is a general response to injury [30], but it has specific consequences in the heart, where it increases myocardial stiffness—a fundamental feature of diastolic dysfunction [29]. DNA methylation has been linked to cardiac fibroblast activation and cardiac fibrosis [31]. Cardiac fibrosis consists of an excessive accumulation of extracellular matrix in the myocardial interstitium [32].

The pathophysiology of cardiac fibrosis can be triggered in response to myocardial insults such as myocardial hypoxia or inflammation. Hypoxia-induced cardiac fibrosis is associated with DNA hypermethylation and increased expression of DNMT1 and DNMT3B [33]. Hypoxia can stimulate human cardiac fibroblasts to express HIF-1α, leading to the activation of pro-fibrotic genes, which has been linked to the upregulation of DNMT1 and DNMT3a/3b [33,34].

DNA methylation has been implicated in the development of cardiac fibrosis. This can occur through various pathways. In cardiac fibroblasts, the hypoxia-induced inactivation of RASSF1A along with the activation of ERK1/2 produces fibroblast proliferation and cardiac fibrosis [35]. Two Ras family members have been implicated in these processes. In the human heart, cardiac fibrosis correlates with aberrant RAS protein activator like 1 (RASAL1) promoter methylation, transcriptional RASAL1 suppression, increased Ras-GTP activity, and increased expression of EndMT markers [36]. These data suggest aberrant Rasal1 promoter methylation and hydroxymethylation are regulatory factors involved in cardiac fibrosis [36]. A reduction in DNMT3A produces an increase in Ras association domain family member 1 (RASSF1A) expression in activated cardiac fibroblasts, which upregulates p-ERK1/2 [37]. Thus, the downregulation of RASSF1A, which can occur through DNA methylation, is associated with cardiac fibrosis and fibroblast activation [37].

DNMT1 is also operative in cardiac fibrosis. DNMT1 hypermethylation reduces the expression of microRNA-152-3p (miR-152-3p) and promotes the Wnt1/beta-catenin signaling pathway, leading to the proliferation and activation of cardiac fibrocytes [38].

**Figure 1:**
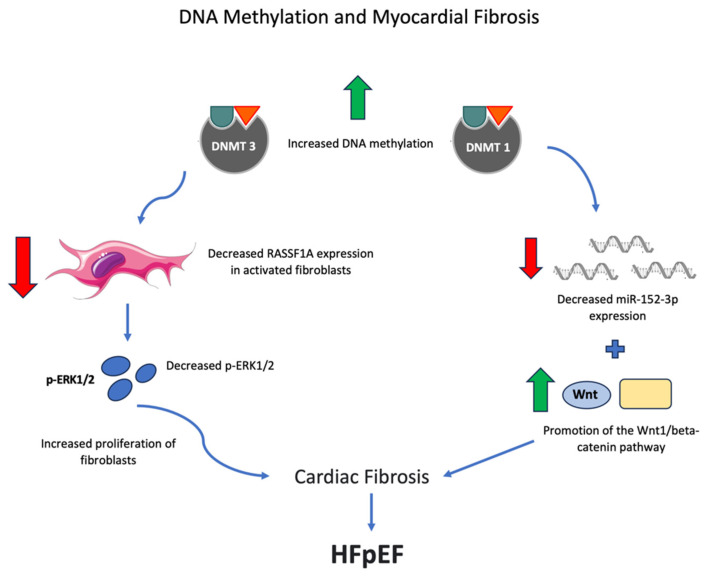
Increased DNA methylation by DNMT1 and DNMT3 leads to changes in fibroblast proteins and miR-152-3p expression, leading to cardiac fibrosis (through various signaling pathways) and the development of HFpEF. Red arrows represent a decrease and green arrows represent an increase. (Fibroblast image: Fibroblasts/Laboratoires Servier/Creative Commons Attribution-Share Alike 3.0 Unported).

Histone lysine-specific demethylase 1 (LSD1) was significantly increased, threefold, in HF from dilated cardiomyopathy [39]. LSD1 was upregulated in Angiotensin II (Ang II)-treated neonatal rat cardiac fibroblasts, which was reversed by LSD1 silence [39]. LSD1 inducible knockout in vivo, in mice, significantly alleviated cardiac fibrosis as well as systolic dysfunction and cardiac hypertrophy, after transverse aortic constriction [39]. The loss of LSD1 in Ang II-induced myofibroblasts involved the intracellular upregulation of TGFβ1 (transforming growth factor β1), its downstream effectors Smad2/3 phosphorylation, as well as the phosphorylation of p38, ERK1/2, and JNK, but also reduced the supernatant TGFβ1 secretion [39]. Some of these relationships can be summarized graphically (Figure 1).

Blockade of DNMT3B expression by siRNA significantly reduced collagen 1, and the DNMT inhibitor (5-aza-2′-deoxycytidine) suppressed the pro-fibrotic effects of TGFβ [31]. 5-aza-2′-deoxycytidine, 5-azacytidine, and some selective histone deacetylase inhibitors can inhibit cardiac fibrosis. [34].

## 5. SR Calcium ATPase

A less frequently considered pathway in the production of HFpEF involves the sarcoplasmic reticulum Ca^2+^-ATPase (SERCA). Cardiac relaxation is regulated by SERCA2a and its isoforms because they are responsible for the majority of calcium reuptake into the sarcoplasmic reticulum (SR) [40,41]. The loss of SERCA2a activity reduces the amount and rate of calcium removal from the cytoplasm into the SR, reducing cardiac relaxation and producing diastolic dysfunction, which underlies HFpEF [42].

**Figure 2:**
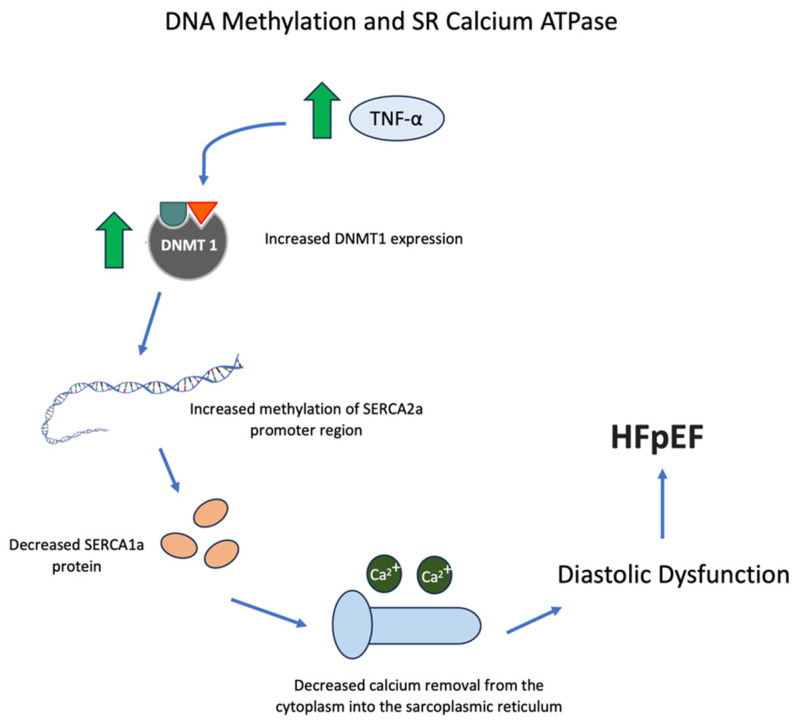
Increased TNF-α leads to increased DNMT1 expression and methylation of the SERCA2a promotor, causing decreased calcium return into the sarcoplasmic reticulum and diastolic dysfunction which is present in HFpEF. Green arrows represent an increase.

Cytokine levels, specifically TNF-α and interleukin-6 (IL-6), correlate with diastolic function in critically ill patients, and diastolic function improves significantly in association with decreased levels of these cytokines [43]. TNF-α and IL-6 are amongst the factors that downregulate SERCA2 gene expression [43,44]. Increased methylation of the SERCA2a promoter region can be induced by TNF-α, which increases the expression of DNA methyltransferase 1, leading to lower levels of SERCA2a RNA and protein in cardiomyocytes [45]. Some of these relationships can be summarized graphically (Figure 2).

It is possible to reverse cardiac dysfunction by increasing SERCA2a through gene transfer [46]. A possible treatment of HFpEF that warrants further investigation is the use of hydralazine. In HL-1 cardiomyocytes, hydralazine increased intracellular Ca^2+^ transients and SR Ca^2+^ contents [47]. Hydralazine decreased the expression of DNA methyltransferases, decreased methylation in the SERCA2a promoter region, and increased the RNA and protein expressions of SERCA2a [47]. In addition, treatment of isoproterenol-induced heart failure rats with hydralazine decreased the promoter methylation of SERCA2a and increased SERCA2a RNA expression [47].

While some animal data suggest that hydralazine plus sodium nitrite significantly attenuated the severity of HFpEF [48], the combination of hydralazine with isosorbide dinitrate in patients with HFpEF appears to have deleterious effects on myocardial remodeling and submaximal exercise, findings that do not support the routine use of hydralazine in patients with HFpEF [49].

## 6. Myocardial Inflammation

Cardiac fibrosis, a fundamental aspect of HFpEF, can be triggered in response to myocardial inflammation [28,29]. The myocardium of patients with HFpEF has inflammatory cells (macrophages) that express the profibrotic growth factor TGF-β [50]. There is an associated accumulation of cardiac collagen and a reduction in matrix metalloproteinase-1 [50]. HFpEF is also associated with increased numbers of CD^3+^, CD11a^+^, and CD45^+^ cells, as well as VCAM-1 [50]. There is a direct correlation between the number of inflammatory cells and the degree of diastolic dysfunction, linking myocardial inflammation to cardiac fibrosis and, consequently, diastolic dysfunction [50].

Inhibition of DNA methylation by 5-azacytidine produces a shift in the type of macrophage to those with an anti-inflammatory phenotype [51]. The effect of DNA methylation inhibition includes the sumoylation of interferon regulatory factor-1 (IRF1) in macrophages [51]. SUMO (small ubiquitin-related modifier) proteins are ~10 kD polypeptides that function as reversible post-translational protein modifiers. They form isopeptide bonds with ɛ-amino groups of acceptor Lys residues in hundreds of target proteins in a process termed sumoylation [52]. Thus, the inhibition of DNA methylation may operate through IRF1 sumoylation to inhibit cardiac fibrosis [51], while DNA methylation accentuates cardiac fibrosis through this mechanism.

Epigenetic changes in cardiac myocytes are associated with heart failure [53]. Heart failure in the Dahl salt-sensitive rat, an animal model of HFpEF [54,55], is linked to increases in the expression of 12/15-LOX [56]. Alox15, which encodes the protein 12/15-LOX, was markedly upregulated in failing hearts compared with control hearts [56]. LOXs are a family of lipid-peroxidizing enzymes that metabolize the oxidation of polyenolic fatty acids into their corresponding hydroperoxy derivatives [57]. Transgenic mice that overexpressed 12/15-LOX manifested increased cardiac fibrosis with macrophage infiltration [56]. Thus, in an animal model of HFpEF with hypertension, 12/15-LOX causes heart failure by promoting cardiac inflammation and fibrosis [56].

**Figure 3:**
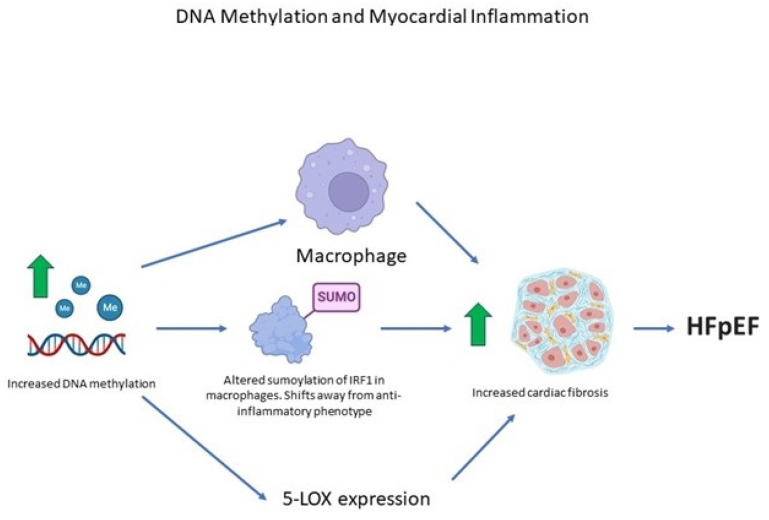
Chronic inflammation has been linked to metabolic stress and chronic inflammation, and each factor separately, as well as together, induces HFpEF [60]. (Some images sourced from biorender.com).

5-lipoxygenase (5-LOX) is the key enzyme in the biosynthesis of leukotrienes, which are pro-inflammatory lipid mediators derived from arachidonic acid [58]. The expression of 5-LOX mRNA in heart tissue is about fifty times greater than in brain tissue [59]. 5-LOX expression is regulated by DNA methylation [56,58,59,60,61]. Some of these relationships can be summarized graphically (Figure 3).

Increased DNA methylation shifts macrophages away from an anti-inflammatory phenotype, leading to increased cardiac fibrosis and HFpEF. Green arrows represent an increase.

Chronic inflammation has been linked to metabolic stress and chronic inflammation, and each factor separately, as well as together, induces HFpEF [62].

## 7. Mitochondrial and Metabolic Defects

**Figure 4:**
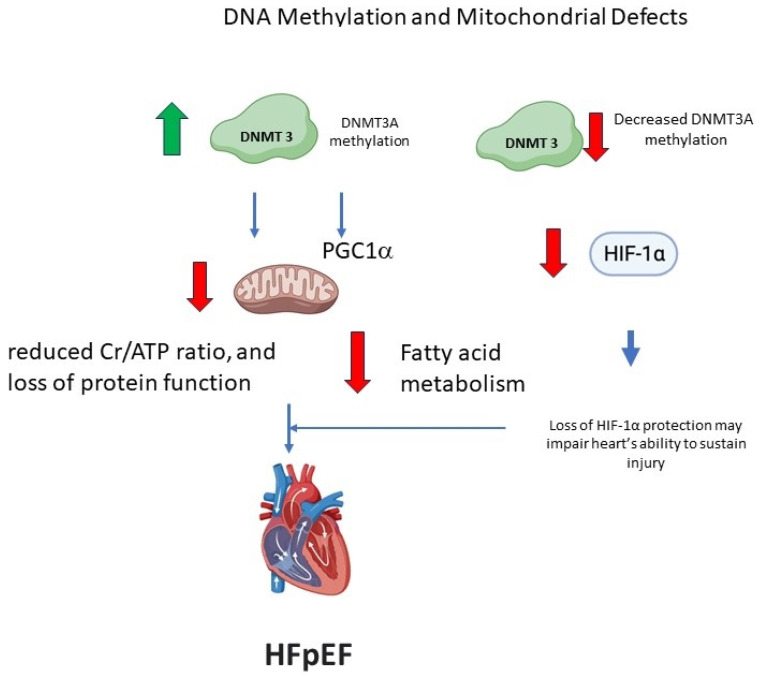
Increases and decreased DNMT3A methylation can have different kinds of impacts on mitochondrial energy metabolism and production of factors that can reduce cardiac function and/or limit the heart’s ability to sustain injury, leading to HFpEF. Red arrows represent a decrease. (Some images sourced from biorender.com).

Mitochondrial dysfunction is purported to be involved in the pathophysiology of HFpEF through a number of different pathways, including increased reactive oxygen species (ROS) production, reduced cardiac creatine phosphate/adenosine triphosphate (ATP) ratio, and loss of normal protein function [63,64,65]. Some of these relationships can be summarized graphically (Figure 4). In a risk model predicting the development of HFpEF, 25 CpGs had key functions related to energy metabolism as well as intracellular signaling [12]. Superoxide dismutate (SOD) significantly attenuates RASSF1A gene methylation and can alleviate cardiac fibrosis induced by hypoxia [33]. Another factor operating in this system is the miR-29 family, which can prevent hypoxia-dependent hypermethylation via downregulation of DNMTs [66].

In HF, the pathways that regulate the elimination of dysfunctional mitochondria are also disrupted, resulting in a buildup of damaged mitochondria and cardiomyocyte death [67]. DNMT3A may be involved in maintaining normal mitochondrial function, structure, and lipid metabolism [21]. DNMT3A was knocked out of human-induced pluripotent stem cells (hiSC) and engineered into heart tissue (EHT) after differentiation into cardiomyocytes [21]. On transmission electron microscopy, abnormal cristae structure, swollen mitochondria, and an accumulation of lipid droplets in pre-apoptotic cells without sarcomeres were observed in KO EHTs vs. wild-type (WT) EHTs. ATP production, basal and maximal respiration, and non-mitochondrial respiration were also significantly lower in the KO cells [21]. KO EHTs had significantly lower levels of mitochondrial DNA (mtDNA) and mRNA transcripts encoded by mtDNA than WT EHTs [21].

However, in an article by Pepin et al. [27], DNMT3A was suggested to increase in HF and differentially hypermethylate genes associated with oxidative phosphorylation, while the promoters of genes involved in glycolysis and other anaerobic metabolic pathways were hypomethylated. DNA methylation can have consequences on cardiomyocyte signal transduction pathways [68]. DNMT3A hypermethylation can decrease fatty acid oxidation occurring in the myocardium and favor the switch to glycolysis, which is consistent with the observed metabolic changes in HFpEF [69]. Despite this, DNMT3A KO EHTs were shown to have lower mRNA transcripts of enzymes involved in glycolysis, including HIF-1α, and only KO EHTs functionally declined in glucose-only serum compared to WT, suggesting impairments in glycolysis [21]. Glycolysis was also impaired in KO EHTs when the serum was depleted of small molecules, and the addition of small molecules such as the basic fibroblast growth factor and endothelial growth factor prevented the functional decline of the KO EHTs and stabilized HIF target gene expression [21]. This suggests that the contrasting results from Pepin et al. [27] may also be explained by other factors besides an increase in DNMT3A.

Heart failure is characterized by a switch in mitochondrial metabolism from the utilization of fatty acids to glucose metabolism, and specifically by an ‘uncoupling’ of glycolysis and pyruvate oxidation by downregulation or inactivation of pyruvate dehydrogenase (PHD) [70,71]. While these metabolic alterations have been characterized best in HFrEF, there is evidence that diastolic dysfunction, the basis of HFpEF, induced by angiotensin II or phenylephrine, is associated with reduced glucose oxidation [72].

Another factor that plays a key role in cellular energy metabolism is the peroxisome proliferator-activated receptor gamma coactivator (PGC-1α), which is a member of a family of transcription coactivators [73]. As a transcriptional coactivator, PGC-1α contains no discernible DNA-binding domain. Moreover, no enzymatic activity has been attributed to this protein. Thus, mechanistically, PGC-1α relies on the selective interaction with transcription factors to be recruited to target genes and subsequently serve as a protein docking platform to recruit other complexes [73]. PGC-1α is a key factor that coordinately regulates the expression of a subset of mitochondrial genes and participates in the overall mitochondrial function in the cell [74]. PGC-1α functions as a coactivator of NRF1, NRF2, and ERR-α/β/γ, which are involved in mitochondrial biogenesis; PPAR-α and δ, which are involved in fatty acid oxidation; TR-β, which is involved in CPT-1 induction; and GR, HNF 4α, and FOXO1, which are involved in gluconeogenesis [73].

The forced expression of PGC-1α in cardiac myocytes induces the expression of nuclear and mitochondrial genes involved in mitochondrial energy transduction/energy production pathways, increased cellular mitochondrial number, and stimulated coupled respiration [75].

The absence of PGC-1α leads to blunted mitochondrial enzymatic activity and decreased levels of ATP in the heart, which is linked to reduced systolic contractile function as well as reduced diastolic function or relaxation (−ve dp/dt) [76]. PGC-1α−/− mice are predisposed to developing heart failure as their hearts display myofibrillar disarray and increased fibrosis [77]. The hypermethylation of PGC-1α decreased the expression of cardiac PGC-1α and induced cardiomyopathy in male rats that was likely operative through the impairment of cardiac mitochondrial function [75]. DNA hypermethylation of PGC-1α contributes to cardiomyopathy in male rats [78].

PGC-1α is sensitive to environmental factors such as environmental chemicals [79]. Thus, it may be one of the pathways whereby environmental factors may play a role in producing HFpEF.

Reduced DNMT3A-mediated methylation in the heart is associated with aberrant activation of the glucose/lipid metabolism regulator peroxisome proliferator-activated receptor gamma, which is associated with an accumulation of lipid vacuoles within cardiomyocytes [21].

Knockout studies of the main cardiac DNMT, DNMT3A, were associated with a lower protein abundance of HIF-1α [21]. As we have summarized previously [80], HIF-1α has been implicated in playing a role in myocardial injury. HIF-1α protects against acute myocardial ischemia reperfusion injury by “promoting aerobic glycolysis, decreasing mitochondrial oxidative stress, activating d hexokinase II, and inhibiting mitochondrial permeability transition pore opening” [81]. The basic fibroblast growth factor may exert its cardioprotective effect by upregulating HIF-1α mRNA in the ischemic myocardium [82]. Thus, an impairment or reduction in DNMT3A-mediated DNA methylation, which produces a reduction in HIF-1α [21], should limit the heart’s ability to sustain injury leading to heart failure. The complexity of the relationships between methylation of key cardiac factors can differentially affect the processes leading to HFpEF. Some of these relationships are illustrated graphically in Figure 4.

## 8. Obesity

Obesity can be a major factor in HFpEF [4,83]. The analysis of HFpEF subtypes by some investigators concluded that there was a specific subtype of HFpEF associated with obesity [4,83]. Obesity is produced by a complex dynamic of energy consumption, expenditure, and metabolism. It can be induced by increased dietary fat intake, which, in animals, is reproduced by feeding a high-fat diet (HFD) [84]. The hearts of mice treated with a high-fat diet demonstrate alterations in the levels of DNA methylation/hydroxymethylation along with decreased mitochondrial mass, a reduction in α-ketoglutarate, and augmented oxidative stress [84]. These changes do not occur in the brain, indicating that the influence of high-fat diets on DNA methylation/hydroxymethylation is not a general process [84].

Obesity is associated with an increase in several inflammatory markers, suggesting that it is a chronic, low-grade inflammation [85]. As discussed above, inflammation in the heart or its surrounding epicardial fat [86] can lead to HFpEF.

**Figure 5:**
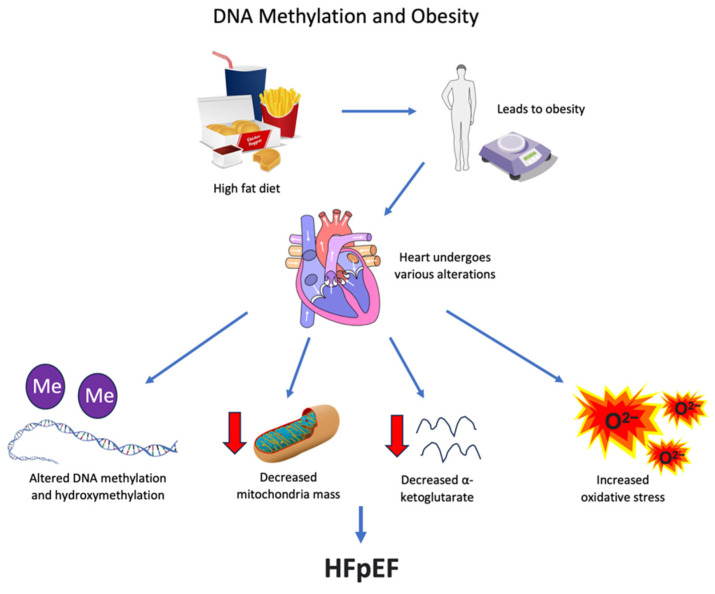
High-fat diets lead to obesity and cause various alterations to the heart, including changes to DNA methylation, mitochondria mass, α-ketoglutarate, and oxidative stress. These pathways can lead to the development of HFpEF. Red arrows represent a decrease.

DNA methylation has been implicated in the pathogenesis of obesity, and the modulation of DNA methylation caused by diet and the environment has also been proposed as a therapeutic strategy to prevent or treat obesity and its complications [87,88]. These relationships can be summarized graphically (Figure 5).

## 9. Aging, Atrial Fibrillation, and Other Comorbidities of Patients with HFpEF

HFpEF is linked to aging, as the incidence of HFpEF increases in older age groups [4,89]. DNA methylation occurs during aging and has been proposed to contribute to the aging process [90,91]. Increased epigenetic age acceleration in whole blood was a risk marker for heart failure independently of chronological age and traditional CVD risk factors [92]. The demonstration of epigenetic deregulation during aging has suggested that “epigenetic rejuvenation” may be an approach to delaying or reversing cardiovascular conditions such as heart failure [92].

Atrial fibrillation has been linked to HFpEF [93,94]. In a multivariate analysis considering age, the left atrial volume index, the E/A and E/e’ ratios, and the left ventricular internal diameter (LVID), only age and the left atrial volume index were significant independent factors related to the presence of atrial fibrillation in HFpEF [94]. DNA methylation has been implicated in the pathogenesis and maintenance of atrial fibrillation [9]. In a rat model of heart failure, the atrium in heart failure induced by isoproterenol manifested increased Pitx2c promoter methylation with increased DNMT 1 and decreased Pitx2c protein levels [95]. Pitx2c can alter the expression of the potassium inward rectifying channel (Kir 2.1), which can lead to arrhythmias [96]. Increased cardiac fibrosis, which has been discussed above, is present in the atrium, and atrial fibrosis is a common feature in the development of atrial fibrillation [97]. Taken together, the increase in DNA methylation in the atrial tissue of patients with atrial fibrillation [9] and DNA methylation in the left ventricle of patients with HFpEF (discussed above) suggests that DNA methylation may be a common mechanism leading to both HFpEF and atrial fibrillation.

There are other factors associated with HFpEF, such as coronary artery disease and diabetes mellitus, that have been linked to DNA methylation [98,99]. Their interrelationship bears further study.

## 10. Conclusions

This review compared studies that investigated the effect of DNA methylation in the heart in order to arrive at a clearer concept of how epigenetic changes can contribute to the pathogenesis of HFpEF. HFpEF is characterized by a complexity of phenomena that, on a global level, are represented by diastolic dysfunction and left ventricle (LV) diastolic stiffness, but on a cellular level, involve alterations to cardiomyocyte elements (mitochondria and SR) and cardiac fibroblasts. We argue that DNA methylation plays a role in HFpEF through its regulatory actions on mitochondrial proteins, which leads to impairments in glycolysis and mitochondrial metabolism, decreased cardiac contractility, and cardiac fibrosis (see graphical summary).

## Data Availability

Data from referenced publications.
